# Gastrointestinal relapse of multiple myeloma and sustained response to lenalidomide: a case report

**DOI:** 10.1186/1752-1947-5-110

**Published:** 2011-03-19

**Authors:** Patrick R Benusiglio, Thomas A McKee, Xavier Montet, Jean-Marc Dumonceau, Laurence Favet, Anne-Claude George, Pierre-Yves Dietrich

**Affiliations:** 1 Centre for Oncology, Geneva University Hospital, 4 rue Gabrielle Perret-Gentil, 1211 Geneva 14, Switzerland; 2 Department of Pathology, Geneva University Hospital, 4 rue Gabrielle Perret-Gentil, 1211 Geneva 14, Switzerland; 3 Department of Radiology, Geneva University Hospital, 4 rue Gabrielle Perret-Gentil, 1211 Geneva 14, Switzerland; 4 Department of Gastroenterology, Geneva University Hospital, 4 rue Gabrielle Perret-Gentil, 1211 Geneva 14, Switzerland

## Abstract

**Introduction:**

Gastrointestinal relapse in patients with multiple myeloma is very rare and, when reported, always associated with a poor prognosis.

**Case presentation:**

We describe the case of a 71-year-old Caucasian man who presented with life-threatening hematemesis and melena due to a digestive relapse of his multiple myeloma. Despite the active hemorrhage, we initiated a third-line treatment with lenalidomide. The response was spectacular and long-lasting.

**Conclusions:**

Clinicians must consider digestive tract involvement in myeloma patients presenting with a gastrointestinal hemorrhage. Furthermore, myeloma patients do benefit from novel oral drugs, even when they are critically ill.

## Introduction

The involvement of the gastrointestinal tract years after an initial diagnosis of multiple myeloma (MM) is exceptional and, when reported, always associated with a poor prognosis [1-4]. We report the case of a 71-year-old man with MM who had been heavily pre-treated and who presented with hematemesis and melena due to a gastrointestinal relapse of his disease. The bleeding lasted for over two weeks and soon became life-threatening. Despite the active hemorrage, we initiated a third-line treatment with lenalidomide. The response was spectacular.

## Case presentation

We report the case of a 71-year-old Caucasian diabetic man with severe diabetic neuropathy who was diagnosed with stage IIIA IgG λ MM in 2004. He was initially treated with three cycles of vincristine, doxorubicin and dexamethasone, followed by high-dose melphalan and autologous stem-cell transplantation, which resulted in a partial response. His monoclonal IgG had dropped from 65 g/L before treatment to 11 g/L after transplantation. In 2007, a second-line chemotherapy treatment (melphalan and prednisone, six cycles) for a relapse characterized by diffuse spinal involvement and an increase in monoclonal IgG (22 g/L) stabilized the disease. His neuropathy had precluded treatment with thalidomide or bortezomib. Eight months after the second-line chemotherapy, an irradiation to the T10-L1 vertebrae (30 Gy) was undertaken for symptomatic, localized bone involvement. Ten months later, an increase in his level of IgG (34 g/L), combined with widespread bone pain and a worsening of his general condition, led to the introduction of high-dose steroids.

**Figure 1:**
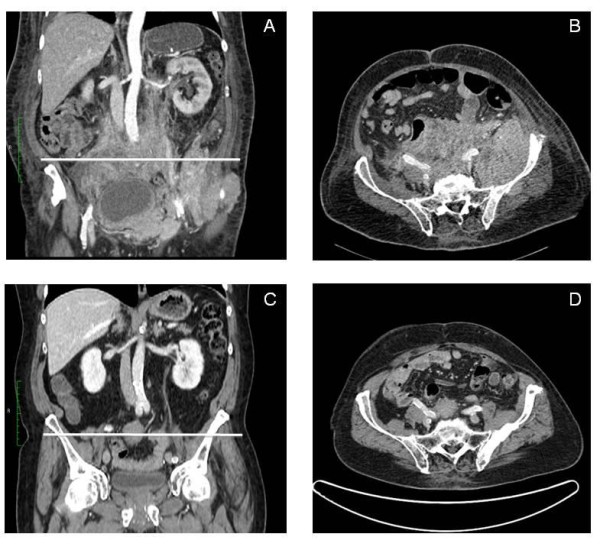
**CT of the abdomen before (A, B) and after (C, D) four months of treatment with lenalidomide**.

After a week of steroid treatment, he was admitted to our hospital for the first time with chest pain and dyspnea. He was febrile (38.4°C) and his inflammatory parameters were increased (C-reactive protein 91 mg/L). A urinary test for the *Legionella pneumophila *antigen was positive and a computed tomography (CT) scan showed trilobar consolidation and a bilateral pleural effusion. A heterogeneous solid mass extending from the retroperitoneal to the peritoneal spaces (Figure 1A,B) provided evidence for the progression of the MM. At the time, priority was given to the treatment of the pulmonary infection and he recovered after three weeks of oral levofloxacin.

**Figure 2:**
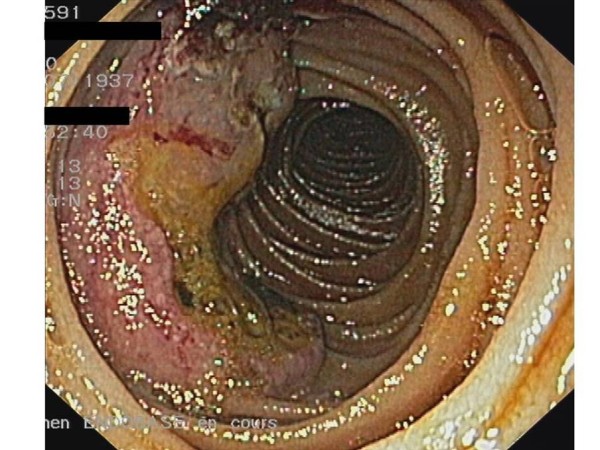
**Jejunal mass as seen on the upper gastrointestinal endoscopy**.

**Figure 3:**
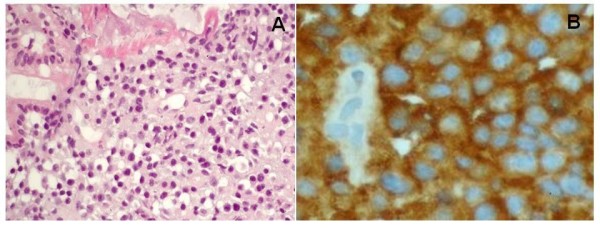
**Jejunal mucosa infiltrated by multiple myeloma**. A: magnification ×200, hematoxylin and eosin. B: magnification ×400, λ chains.

Shortly after the antibiotic therapy was discontinued, he presented with sudden hematemesis and melena, requiring fifteen 500 ml units of packed red cells, in total, over a period of twenty days. His platelet count and coagulation parameters were normal. A bleeding, ulcerated jejunal mass was revealed by an upper gastrointestinal endoscopy (Figure 2) and biopsies showed an infiltration of the intestinal mucosa by neoplastic plasma cells producing monoclonal λ light chains (Figure 3A,B). Despite the active bleeding, a third-line therapy with lenalidomide (25 mg daily) and dexamethasone (40 mg once-weekly) was initiated; the lenalidomide was given in three-week cycles followed by a one-week break [5,6]. An excellent response was achieved after the first cycle: his paraprotein levels dropped to 10 g/L and there was no recurrence of the hematemesis or melena. His general condition improved rapidly and he was discharged after the second cycle had commenced. A repeat CT four months later showed a dramatic shrinkage of the retroperitoneal mass (Figure 1C,D). This response lasted for a total of 10 months and resulted in an excellent quality of life for the patient during the whole period. He declined further treatment at retroperitoneal progression and died a few weeks later.

## Discussion

Gastrointestinal involvement in MM is very rare. It most often occurs in the context of an isolated, primary, extramedullary plasmacytoma [7]. Patients with newly-diagnosed MM rarely present with symptoms related to gastrointestinal involvement [8]. A gastrointestinal relapse in patients with long-term MM, such as that observed in the case of our patient, is exceptional. Amongst a total of 553 patients with MM included in two large European studies, 87 experienced an extramedullary relapse but none of these involved the gastrointestinal tract [1,2]. Only one out of six extramedullary relapses reported by a North American Institution involved the gastrointestinal tract [3]. All of these cases had a poor prognosis, with a maximal survival rate of 106 days from diagnosis. Finally, Dawson *et al. *reported the case of a 60-year-old patient with MM with hematemesis, melena and gastroduodenal mucosal lesions [4]. The patient died two weeks after presentation. The gastrointestinal lesions were not biopsied, but their myelomatous nature was likely, as a biopsy of a right breast mass provided pathological evidence of an extramedullary relapse.

## Conclusion

Our case report is well documented and highly informative. It reminds us that, in addition to much more common causes (for example, ulcers), clinicians must consider digestive tract involvement in patients with MM presenting with a gastrointestinal hemorrhage. It also shows that patients with MM who have been heavily pre-treated can benefit from novel drugs, even when they are critically ill. We suggest that the major clinical improvement has to be linked to lenalidomide, since high-dose steroids had been ineffective in this case. Our patient's recovery and the drop in his monoclonal IgG were very rapid. This effect of lenalidomide has already been observed in other life-threatening situations associated with MM, such as severe renal impairment or high-output heart failure secondary to intramedullary arteriovenous fistulas [9,10]. Finally, it should be emphasized that a response to this oral drug was obtained despite active bleeding in the upper digestive tract.

## Consent

Written informed consent was obtained from the patient's next-of-kin for publication of this case report and any accompanying images. A copy of the written consent is available for review by the Editor-in-Chief of this journal.

## Competing interests

The authors declare that they have no competing interests.

## Authors' contributions

PRB, JMD, LF, ACG and PYD were directly involved in the management of the patient. PRB wrote the manuscript with support from TAM and PYD. TAM and XM reviewed and interpreted the pathology slides and CT scan images, respectively. All authors read and approved the final manuscript.
